# Exploring xylose metabolism in non-conventional yeasts: kinetic characterization and product accumulation under different aeration conditions

**DOI:** 10.1093/jimb/kuae023

**Published:** 2024-06-27

**Authors:** Bruna C Bolzico, Sofia Racca, Jorge N Khawam, Rodrigo J Leonardi, Ariel H Tomassi, Maria T Benzzo, Raul N Comelli

**Affiliations:** Grupo de Procesos Biológicos en Ingeniería Ambiental (GPBIA), Facultad de Ingeniería y Ciencias Hídricas (FICH), Universidad Nacional del Litoral (UNL), Ciudad Universitaria, CC 242 Paraje El Pozo, Santa Fe 3000, Argentina; Consejo Nacional de Investigaciones Científicas y Técnicas (CONICET), Ciudad Universitaria, CC 242 Paraje El Pozo, Santa Fe 3000, Argentina; Grupo de Procesos Biológicos en Ingeniería Ambiental (GPBIA), Facultad de Ingeniería y Ciencias Hídricas (FICH), Universidad Nacional del Litoral (UNL), Ciudad Universitaria, CC 242 Paraje El Pozo, Santa Fe 3000, Argentina; Grupo de Procesos Biológicos en Ingeniería Ambiental (GPBIA), Facultad de Ingeniería y Ciencias Hídricas (FICH), Universidad Nacional del Litoral (UNL), Ciudad Universitaria, CC 242 Paraje El Pozo, Santa Fe 3000, Argentina; Grupo de Procesos Biológicos en Ingeniería Ambiental (GPBIA), Facultad de Ingeniería y Ciencias Hídricas (FICH), Universidad Nacional del Litoral (UNL), Ciudad Universitaria, CC 242 Paraje El Pozo, Santa Fe 3000, Argentina; Consejo Nacional de Investigaciones Científicas y Técnicas (CONICET), Ciudad Universitaria, CC 242 Paraje El Pozo, Santa Fe 3000, Argentina; Grupo de Procesos Biológicos en Ingeniería Ambiental (GPBIA), Facultad de Ingeniería y Ciencias Hídricas (FICH), Universidad Nacional del Litoral (UNL), Ciudad Universitaria, CC 242 Paraje El Pozo, Santa Fe 3000, Argentina; Grupo de Procesos Biológicos en Ingeniería Ambiental (GPBIA), Facultad de Ingeniería y Ciencias Hídricas (FICH), Universidad Nacional del Litoral (UNL), Ciudad Universitaria, CC 242 Paraje El Pozo, Santa Fe 3000, Argentina; Grupo de Procesos Biológicos en Ingeniería Ambiental (GPBIA), Facultad de Ingeniería y Ciencias Hídricas (FICH), Universidad Nacional del Litoral (UNL), Ciudad Universitaria, CC 242 Paraje El Pozo, Santa Fe 3000, Argentina; Consejo Nacional de Investigaciones Científicas y Técnicas (CONICET), Ciudad Universitaria, CC 242 Paraje El Pozo, Santa Fe 3000, Argentina

**Keywords:** Xylose utilization, Non-conventional yeasts, Bioethanol, Xylitol, Kinetic characterization

## Abstract

d-Xylose is a metabolizable carbon source for several non-*Saccharomyces* species, but not for native strains of *S. cerevisiae*. For the potential application of xylose-assimilating yeasts in biotechnological processes, a deeper understanding of pentose catabolism is needed. This work aimed to investigate the traits behind xylose utilization in diverse yeast species. The performance of 9 selected xylose-metabolizing yeast strains was evaluated and compared across 3 oxygenation conditions. Oxygenation diversely impacted growth, xylose consumption, and product accumulation. Xylose utilization by ethanol-producing species such as *Spathaspora passalidarum* and *Scheffersomyces stipitis* was less affected by oxygen restriction compared with other xylitol-accumulating species such as *Meyerozyma guilliermondii, Naganishia liquefaciens*, and *Yamadazyma* sp., for which increased aeration stimulated xylose assimilation considerably. *Spathaspora passalidarum* exhibited superior conversion of xylose to ethanol and showed the fastest growth and xylose consumption in all 3 conditions. By performing assays under identical conditions for all selected yeasts, we minimize bias in comparisons, providing valuable insight into xylose metabolism and facilitating the development of robust bioprocesses.

**One-Sentence Summary:**

This work aims to expand the knowledge of xylose utilization in different yeast species, with a focus on how oxygenation impacts xylose assimilation.

## Graphical Abstract

**Graphical Abstract:**
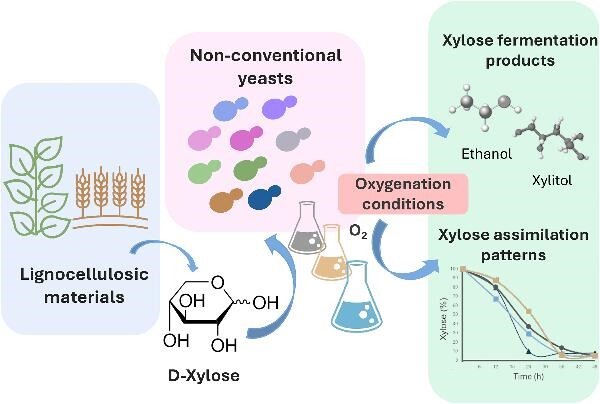

## Introduction

d-Xylose is the second most prevalent monosaccharide in lignocellulosic biomass, after glucose. This C5 carbohydrate is an important component of the plant cell wall hemicellulose, with values varying up to 30% depending on the type of plant biomass. Since lignocellulosic materials represent a promising and sustainable alternative for second-generation bioethanol (2G) production, several investigations have been focused on the metabolic conversion of xylose to ethanol by yeasts (Basso et al., 2023). Successful utilization of xylose would lead to a feasible and cost-effective biomass-to-bioethanol process by increasing the ethanol titer in the fermentation of xylan-rich materials. Although *Saccharomyces cerevisiae* is the best-characterized ethanol-producing microorganism and is widely used to ferment feedstocks containing glucose, fructose, and/or sucrose, this yeast cannot naturally utilize xylose. Significant efforts have been made to genetically modify this yeast. Approaches include the introduction of two different xylose catabolic routes: the xylose isomerase (XI) and the oxidoreductive xylose reductase/xylose dehydrogenase (XR/XDH) pathways, mostly expressed separately (Cunha et al., 2020), but also simultaneously with clear advantages when using non-detoxified lignocellulosic hydrolysates as a substrate (Cunha et al., 2019). In addition, the implementation of specific xylose transporter proteins from other microorganisms has been pursued (Bueno et al., 2020). Furthermore, a redesign of the endogenous hexose transporters has also been applied to increase the affinity and uptake of xylose. Still, with the sole insertion of these pathways and transporter proteins, the xylose fermentation rates of recombinant *S. cerevisiae* strains are lower than those shown on glucose, the preferred substrate. A number of bottlenecks still need to be overcome, including *i)* transport kinetics and glucose inhibition on xylose uptake, *ii)* internal signaling and the lack of a specific sensory machinery to respond rapidly to the sugar, and *iii)* glucose regulatory pathways in the presence of xylose (Brink et al., 2021). In this scenario, consolidated bioprocessing (CBP) systems have been identified as the most promising fermentation configuration for bioethanol production from lignocellulosic biomass. The CBP approaches involve the application of *i)* a microbial consortium of ethanol-producing and hydrolytic enzyme-secreting microorganisms, *ii)* a native strain capable of producing enzymes and fermenting pentoses and hexoses, and *iii)* fermentative and enzyme-secreting engineered strains (Periyasamy et al., 2023).

Several non-*Saccharomyces* yeasts are recognized for their natural ability to efficiently assimilate xylose as a primary carbon source. This trait positions them as a potential biocatalytic platform to use in CBP of 2G bioethanol production (Ndubuisi et al., 2023). Given their ecological diversity and different metabolic profiles, the isolation of new species and strains and the appropriate physiological characterization of native xylose-consuming yeasts are of particular importance (Campos et al., 2022; Cadete & Rosa, 2018). According to their unique metabolic capacities and susceptibility to the variables of the fermentation process, in which oxygenation is an important factor, these yeasts will produce different amounts of ethanol and/or xylitol. Examples of yeasts that have the potential to ferment xylose to ethanol include *Scheffersomyces* (*Pichia*) *stipitis, Pachysolen tannophilus*, and *Spathaspora passalidarum* (Cadete et al., 2016). Instead, the bioconversion of xylose to xylitol by yeasts has been usually attributed to *Candida* species, including *Meyerozyma* (formerly *Candida*) *guilliermondii* and *C. tropicalis* (Kim et al., 2019), among others (Baptista et al., 2021).

Understanding xylose consumption and metabolism in native yeasts is crucial for the development of robust bioprocesses. Oxygen has an important influence in xylose metabolic reactions: while high aeration promotes growth, oxygen limitation favors the production of metabolites such as ethanol. Therefore, it is imperative to determine the optimal oxygenation setup to achieve the highest efficiency in xylose fermentation to ethanol. Moreover, knowledge of the physiological responses under different oxygen conditions can be useful to elucidate how the metabolism of pentose is regulated in different yeast genera. Overall, this understanding can help redirect novel or underutilized resources, such as sugars from industrial wastewater or lignocellulosic feedstocks, toward the production of desired metabolites like ethanol. In terms of xylose usage, the majority of available studies have focused on describing the performance of the most commonly reported xylose-fermenting yeasts: *Sp. passalidarum* and *Sc. stipitis* (Bonan et al., 2021). However, there is limited information on the performance of other xylose-metabolizing yeast species. Additionally, the documented fermentation and oxygenation conditions used to evaluate yeasts vary across the literature, limiting the comparability of results. Therefore, a larger number of yeasts assessed under the same experimental conditions are still required.

This work aimed at evaluating and comparing the performance of selected native xylose-assimilating yeasts from diverse genera. These evaluations were performed through assays using xylose as the sole carbon source under various aeration conditions. A comparative analysis of xylose utilization and product accumulation was conducted using both newly isolated strains (*Kluyveromyces marxianus, Yamadazyma* sp., *M. guilliermondii, Meyerozyma* sp., and *Naganishia liquefaciens*) and type xylose-fermenting yeasts (*Sp. passalidarum, Sc. stipitis*, and *P. tannophilus*). A recombinant strain of *S. cerevisiae*, constructed by chromosomal integration of the *Sc. stipitis* genes encoding d-xylose reductase (XR), xylitol dehydrogenase (XDH), and overexpression of xylulokinase (XK) (Wahlbom et al., 2003), was included for comparative purposes. Kinetic parameters, xylose consumption, and metabolite yields were estimated to explore distinctive features in xylose-metabolizing yeasts that could lead to their utilization in a CBP configuration.

## Materials and Methods

### Strains and Media

The yeast strains employed in this study are listed in Table 1. All strains were maintained in 30% (v/v) glycerol at −80 °C. *Saccharomyces cerevisiae* TMB3400 was kindly provided by Marie F. Gorwa-Grauslund (Lund University, Sweden). A group of xylose-consuming yeasts were isolated from environmental samples collected in Santa Fe, Argentina. The samples were suspended in YPX medium (yeast extract 5g⋅L^−1^, peptone 3 g⋅L^−1^, and d-xylose 20 g⋅L^−1^) supplemented with chloramphenicol to inhibit bacterial growth and incubated for 48–72 hr at 30 °C. A loop of the culture was streaked onto solid YPX medium containing 1.5% of agar and the plates were incubated at 30 °C until colonies developed. Isolates were subsequently cultured on Petri dishes containing YPX medium for colony purification. Identification of isolated yeasts was performed through amplification and sequencing of the ribosomal internal transcribed spacer (ITS) and D1/D2 region of the rDNA, using ITS1 and NL4 primers. To search for sequence similarities and identify the genus and species of the isolated strains, the NCBI Blast tool (http://www.ncbi.nlm.nih.gov/) was used.

**Table 1.** Yeast Species Used in This Study

**Yeast**	**Source**	**References**
*Scheffersomyces stipitis* NRRL Y-7124*Pachysolen tannophilus* NRRL Y-2460*Spathaspora passalidarum* NRRL Y-27907	Decaying wood and wood-eating insect guts Wood extracts used in leather tanning Wood-boring beetles	du Preez et al., 1986 Slininger et al., 1982 Nguyen et al., 2006
*Kluyveromyces marxianus*	Dairy wastewater	
*Meyerozyma guilliermondii*	Wine spoilage	
*Meyerozyma* sp.	Tannery wastewater	This work
*Yamadazyma* sp.	Decay wood	
*Naganishia liquefaciens*	Tannery wastewater	
*Saccharomyces cerevisiae* TMB3400	n.a.	Wahlbom et al., 2003

*Note.* Identification of isolated yeasts was based on internal transcribed spacer (ITS) region and D1/D2 domains of rDNA. n.a. = not applicable.

### Fermentation Assays

Prior to fermentation experiments, an inoculum of each species was prepared by culturing frozen cells in YPX medium for 18–24 hr at 30 °C. The cells were then transferred to a 250-ml Erlenmeyer flask containing 50 ml of YPX medium. Cells were cultured at 30 or 35 °C on a rotary shaker at 150 rpm for 48–72 hr, depending on the species. After cultivation, the cells were harvested by centrifugation at 3500 rpm for 5 min, washed twice, and resuspended in sterile distilled water to prepare the final inoculum for fermentation. Three configurations of the fermentation assays were run in batch mode using 100-ml reactors. Biomass growth, xylose consumption, and product accumulation were assessed under different oxygen availability conditions: *a)* In the aerated experiments, air was supplied continuously at a rate of 1.67 vvm (working volume of 30 ml and air flow of 0.05 L⋅min^−1^). The other two conditions involved cultures without air sparging, where the reactors were closed with cotton plugs. These conditions differed in the headspace of the reactors: *b)* reactors with 70% headspace containing 30 ml of working volume (referred to as 70% HS) and *c)* with 40% headspace containing 60 ml of working volume (referred to as 40% HS). The initial yeast OD_600nm_ was adjusted to 1.0 and 10 g⋅L^−1^. d-Xylose was used as the sole carbon source. The medium was supplemented with 5 g⋅L^−1^ of commercial yeast extract and the pH was adjusted to 5.50 ± 0.10. The flasks were incubated at a constant temperature of 35 °C for the different yeast species and 30 °C for *N. liquefaciens*. This was because the majority of *Naganishia* species, including *N. liquefaciens*, only tolerate growth temperatures at or below 30 °C (Kurtzman et al., 2010). Strictly anaerobic cultivations were avoided as many non*-Saccharomyces* yeast species are unable to perform xylose fermentation in the complete absence of oxygen. In configurations *b)* and *c)*, yeast growth on xylose was supported by the residual oxygen in the headspace of the reactors, the amount of air diffused through the cotton plug and the continuous shaking during fermentation. In all cases, the reactors were shaken at 120 rpm to avoid biomass precipitation. The initial fermentation time was set upon reactor inoculation and all fermentation assays were stopped 24 hr after inoculation. Xylose consumption and yeast growth were monitored over time with sampling intervals of either 2 or 4 hr depending on the culture conditions. Sample volumes were adjusted to ensure that at least 60% of the initial volume remained in the flasks at the end of the experiment. Several parameters were determined to evaluate the performance of each strain (Comelli et al., 2020). All fermentation experiments are presented as averages of biological duplicates.

### Analytical Measurements

Aliquots of the culture were taken at different intervals and centrifuged at 1200 *g* for 5 min. The supernatants were transferred to new tubes and stored at −20 °C until the appropriate determination. The precipitated cells were washed twice with distilled water and resuspended in the starting volume. Optical density (OD) was measured at 600 nm using a VIS spectrophotometer (DR/2010, HACH, USA). d-Xylose and xylitol quantification were performed using high-performance liquid chromatography (UltiMate 3000 HPLC system, ThermoFisher, USA) coupled to a refractive index detector. Separation was conducted using the Hypersyl APS-2 Amino Column (ThermoFisher, USA) at a column temperature of 30 °C. The mobile phase consisted of 83% acetonitrile and 17% water, with a flow rate of 1.250 ml⋅min^−1^. Ethanol was quantified using a gas chromatograph (GC-2014 system, Shimadzu, USA) equipped with a flame ionization detector and the TR-Wax GC column (ThermoFisher, USA). The injector and the detector temperatures were set to 240 and 250 °C, respectively, and hydrogen was used as the gas carrier. The column oven temperature was initially set to 40 °C and held for 0.5 min before being ramped up at 5° C⋅min^−1^ to a final temperature of 80 °C, which was held for 0.50 min.

### Phylogenetic Tree Analysis

The relationship between the genera and species of yeasts isolated in this work and known xylose-utilizing yeasts was established by constructing phylogenetic trees. These trees were based on ITS/D1-D2 regions of the rDNA and amino acid sequences of XR and XDH (*XYL1* and *XYL2* encoding genes, respectively). Multiple alignment and phylogenetic tree construction were performed using available sequences (accession numbers in Supplementary Table S1). The corresponding sequences of *Rhizopus arrhizus* were used as the outgroup. Multiple sequence alignments were generated for each set of sequences using MUSCLE (Edgar, 2004) with the default settings. Phylogenetic analyses were performed using the maximum likelihood method and the general time reversible model for the ITS/D1-D2 regions and the Le_Gascuel_2008 model (Le & Gascuel, 2008) for the XRs/XDHs proteins in MEGA 11 (Tamura et al., 2021). The trees with the highest log likelihood are presented. The percentage of trees in which the associated taxa clustered together in the 1000 bootstrap replicates test is indicated next to the branches. This analysis included 14 nucleotide sequences for ITS/D1-D2 totaling 1665 positions in the final dataset, and 12 amino acid sequences for XR and XDH proteins totaling 331 and 403 positions, respectively, in the final dataset.

## Results

### Isolation and Identification of d-Xylose-Consuming Yeasts

A total of 52 yeasts were previously isolated from agro-industrial wastewater and environmental samples collected in the region of Santa Fe, Argentina (31°02′02.40′ S, 60°41′16.80′ W), and were screened for their ability to metabolize pentoses, mainly xylose and arabinose. Among them, five yeast species demonstrated exceptional performance, exhibiting high rates of xylose consumption and biomass growth, minimal latency time, and complete utilization of xylose, among other criteria (data not shown).

Analysis of the ITS regions and D1/D2 domains facilitated the classification of the five best performing isolates into the following genera: *Kluyveromyces, Meyerozyma, Naganishia*, and *Yamadazyma* (Table 1). Three out of the five isolated strains were identified at the species level: *K. marxianus, M. guilliermondii*, and *N. liquefaciens*. This confirmation was reached after obtaining a sequence identity match of greater than 99% with reference sequences deposited in the NCBI database. The other two species, belonging to the genera *Yamadazyma* and *Meyerozyma*, could not be definitively identified. Consequently, they are referred to as *Yamadazyma* sp. and *Meyerozyma* sp. Additional taxonomic and sequencing analyses are required to accurately identify these strains. In the case of *Meyerozyma* sp., no distinction could be made between *M. carpophila* and *M. caribbica* species because an identical number of mismatches were found in the ITS/D1-D2 regions of all three species. Similarly, the resulting *Yamadazyma* strain sequencing was insufficient to distinguish between *Y. terventina* and *Y. mexicana*, the closest species regarding sequence similarity.

The yeasts isolated here were previously reported to be natural xylose consumers (Kurtzman et al., 2010). *Kluyveromyces* is the leading yeast genus with significant industrial potential, as it has been supported by studies in the literature on various aspects of its metabolism (Baptista & Domingues, 2022; Karim et al., 2020), followed closely by *Meyerozyma* and the less explored *Yamadazyma.* However, despite their exploitable industrial applications, the study of xylose metabolism in these genera remains relatively limited. What is more, *Naganishia* is presented as a genus that has received limited research attention, particularly concerning pentose assimilation. *Naganishia liquefaciens*, along with *Meyerozyma* sp., was isolated from recalcitrant tannery wastewaters. This ecological niche resembles that of *P. tannophilus*, one of the earliest strains known for its ability to produce ethanol from xylose (Slininger et al., 1982). Moreover, it offers an opportunity to investigate the activity and characteristics of putative lignin(tannin)-degrading enzymes detected in preliminary tests (data not shown). As this aspect is out of the scope of the present work, future research could explore this feature of the yeasts isolated from tannery wastewaters.

### Comparison of Yeasts Performance on Xylose as a Sole Carbon Source

Three reactor configurations were chosen to study the performance on xylose of different native xylose-consuming yeasts. The conditions included supplying external air at a rate of 1.6 vvm and varying the starting media volume, which differed in the percentage of flask headspace (70% and 40%). In an attempt to analyze xylose consumption profiles, strictly anaerobic or anoxic cultures were avoided because xylose-assimilating yeasts typically do not grow or consume considerable amounts of the pentose under severe oxygen limitations. Also, the production of ethanol from xylose has primarily been associated with microaerobic conditions (Silva et al., 2012). Furthermore, an initial xylose concentration of 10 g⋅L^−1^ was selected for the experiments in order to effectively characterize and compare xylose utilization profiles over time and to avoid limitations related to product inhibition.

The literature suggests that the selected yeasts are Crabtree-negative. However, since studies often employ glucose as the carbon source, we decided to be cautious when switching to xylose. Therefore, we chose a 1:10 inoculum-to-substrate ratio to avoid potential metabolic checkpoints that could alter carbon fate in response to fluctuations in oxygen levels (Dickinson & Schweizer, 2004).

**Fig. 1.**
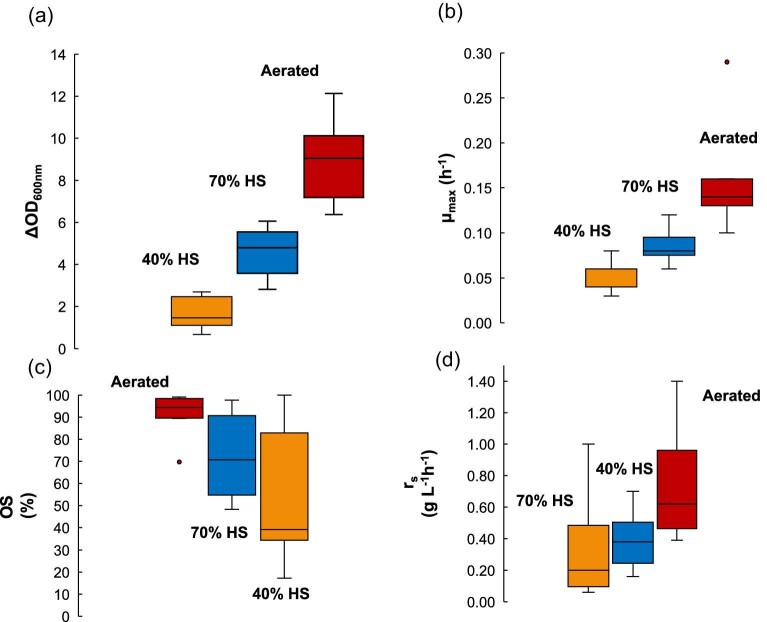
Variation of the parameters total biomass production (a), maximum specific growth rate (b), net xylose consumption (c) ,and maximum xylose consumption rate (d) under different fermentation conditions. Overall effect on the performance of the yeast species on xylose of the three types of reactor design: aerated reactors, 40% headspace (HS),and 70% HS. The data set includes the nine species of yeast that were evaluated.

The performance of the strains, as indicated by the maximum specific growth rate (µ_max_), the total biomass formation (in terms of ΔOD_600nm_), the xylose consumption (ΔS) and the maximum xylose consumption rate (r_s_), differed significantly as a function of the reactor configuration, as depicted in the boxplots in Fig. 1. Overall, most of the d-xylose was converted into biomass during aeration. Under this condition, all the strains showed the highest values of specific growth rate and total biomass formation, which can be attributed to sugar respiration. In line with this, the majority of the xylose (between 90% and 100%) was utilized in the aerated condition (Fig. 1c). Air supply also resulted in faster xylose consumption at the highest maximum rates for most strains compared to the other two conditions (Fig. 1d), with exceptions observed for *K. marxianus, Meyerozyma* sp., and *S. cerevisiae* TMB3400 (see Supplementary Fig. S1). Regarding changes in the media volume without external air provision, the 70% headspace (HS) condition resulted in intermediate values of the parameters related to yeast growth. A reduction in the reactor HS from 70% to 40% led to a significant decrease in the maximum specific growth rates and the biomass production by all the yeast strains (Fig. 1a and b; 40% HS condition). This suggests that in the non-aerated reactors, as the working volume is increased or the headspace is reduced, oxygen availability becomes more limited, resulting in less biomass formation and the lowest values of maximum specific growth rates (Table 2). Moreover, the variations in xylose utilization were more pronounced between the aerated reactors and the 40% HS reactors (Fig. 1d) for most of the strains, which had the highest sugar consumption rates with air supply and the lowest rates in the most oxygen-restricted condition (Table 3).

**Table 2.** Comparison of Yeast Performance in Three Different Culture Conditions Based on Biomass Growth Parameters

	**Parameters** ^a^					
	**Aerated**		**70% headspace**		**40% headspace**	
**Yeast strain**	**µ_max_** **(hr^−^^1^)**	**ΔOD_600nm_**	**µ_max_** **(hr^−^^1^)**	**ΔOD_600nm_**	**µ_max_** **(hr^−^^1^)**	**ΔOD_600nm_**
*Scheffersomyces stipitis*	0.15	9.37	0.07	5.78	0.06	2.26
*Pachysolen tannophilus*	0.13	9.85	0.06	5.32	0.05	2.70
*Spathaspora passalidarum*	0.29	12.14	0.09	4.02	0.08	1.47
*Kluyveromyces marxianus*	0.14	9.04	0.08	4.19	0.06	1.45
*Naganishia liquefaciens*	0.14	6.58	0.10	4.80	0.04	0.67
*Yamadazyma* sp.	0.16	10.39	0.08	3.14	0.03	1.00
*Meyerozyma guilliermondii*	0.10	9.03	0.09	5.10	0.04	2.67
*Meyerozyma* sp.	0.13	6.37	0.12	6.06	0.04	1.62
*Saccharomyces cerevisiae* TMB3400	0.16	7.78	0.08	2.82	0.04	1.22

*Note.* µ_max_ = maximum specific growth rate; ΔOD_600n_ = net optical density at 600 nm.

^a^µ_max_ values were calculated from the exponential phase of biomass growth for each yeast species.

**Table 3.** Comparison of Yeast Performance in Three Different Culture Conditions Based on Xylose Consumption Parameters

	**Parameters** ^a^					
	**Aerated**		**70% headspace**		**40% headspace**	
**Yeast strain**	**ΔS** **(%)**	**r_s_** **(g⋅L^−^^1^⋅hr^−^^1^)**	**ΔS** **(%)**	**r_s_** **(g⋅L^−^^1^⋅hr^−^^1^)**	**ΔS** **(%)**	**r_s_** **(g⋅L^−^^1^⋅hr^−^^1^)**
*Scheffersomyces stipitis*	100.00	1.17	89.92	0.40	84.98	0.34
*Pachysolen tannophilus*	69.73	0.50	49.33	0.16	36.28	0.19
*Spathaspora passalidarum*	100.00	1.40	97.76	0.70	100.00	1.00
*Kluyveromyces marxianus*	89.70	0.39	70.19	0.38	37.90	0.20
*Naganishia liquefaciens*	98.18	0.62	87.52	0.45	39.20	0.09
*Yamadazyma* sp.	96.46	0.75	53.86	0.23	32.43	0.06
*Meyerozyma guilliermondii*	94.45	0.62	60.96	0.28	17.23	0.10
*Meyerozyma* sp.	93.90	0.45	91.65	0.56	55.74	0.51
*Saccharomyces cerevisiae* TMB3400	89.38	0.48	57.77	0.26	80.72	0.46

Note. ΔS = net xylose consumption; rs = volumetric xylose consumption rate.

^a^r_s_ values were calculated from the exponential phase of xylose consumption for each yeast species.

**Fig. 2.**
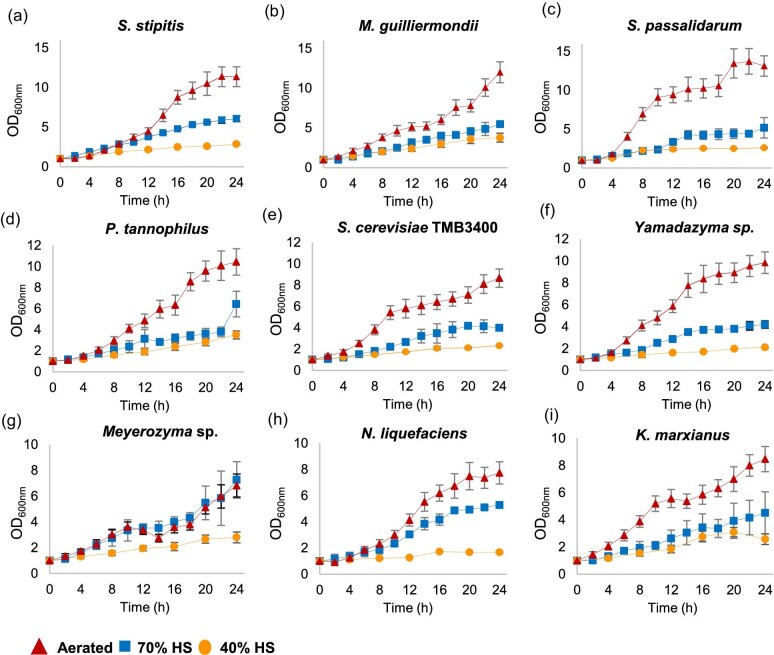
Biomass growth profile on xylose of the nine species considered (a–i) under the three cultivation conditions. Optical density at 600 nm was monitored over time in aerated reactors (triangle) and in non-aerated reactors with 70% (square) and with 40% headspace (circle).

**Fig. 3.**
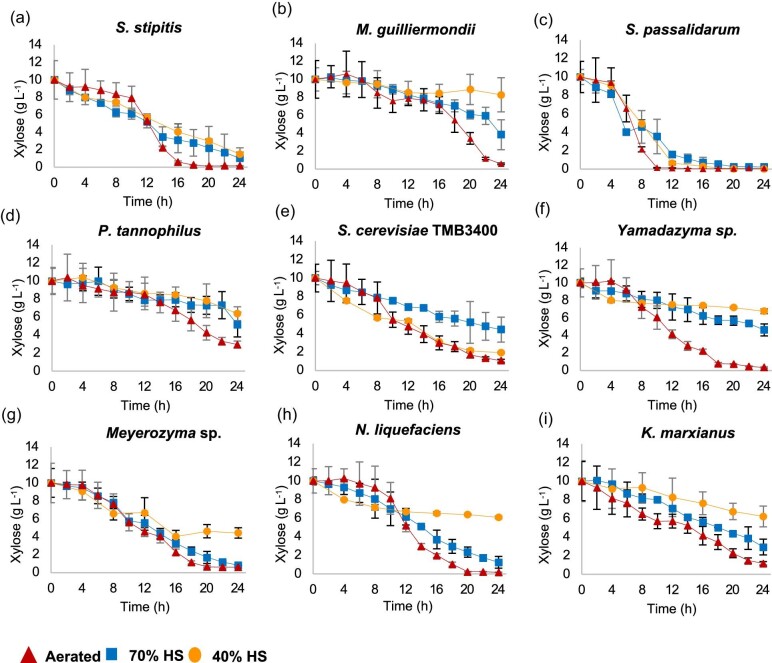
Xylose consumption profile of the nine yeast species considered (a–i). Xylose concentration was monitored over time during the three different cultivation conditions. Aerated reactors (triangle) and non-aerated reactors with 70% (square) and 40% headspace (circle).

Growth and sugar consumption curves (Fig. 2 and Fig. 3, respectively) varied according to the yeast strain and the experimental conditions. The figures clearly show distinct profiles of xylose consumption and biomass accumulation over time for the nine yeasts studied in the three fermentation setups. *Spathaspora passalidarum* produced the highest biomass and reached the highest maximum specific growth rate of 0.29 hr^−1^ under oxygen provision (Fig. 2c), while the µ_max_ values for the other strains (Table 2) ranged from a minimum of 0.10 hr^−1^ for *M. guilliermondii* to 0.16 hr^−1^ for *Yamadazyma* sp. and *S. cerevisiae* TMB3400 in the same condition. The biomass produced was almost two to three times lower when cells were grown in the 70% HS reactors compared to the aerated ones, except for *Meyerozyma* sp., which produced a similar amount of biomass at a similar maximum specific growth rate of 0.12 hr^−1^ in the two conditions. In the most limited oxygen setup (40% HS), the highest value of µ_max_ (0.08 hr^−1^) was reached by *Sp. passalidarum* and the strains produced four to nine times less biomass than in the aerated reactors and between two to three times less biomass than in the 70% HS reactors. *Scheffersomyces stipitis, Sp. passalidarum, P. tannophilus*, and *K. marxianus* showed similar maximum specific growth rates in the 70% and 40% HS conditions, despite the differences in net biomass formation (Table 2).

When external air was supplied, the highest rates of xylose consumption were achieved by *Sp. passalidarum* and *Sc. stipitis.* Among all the yeast species, the former exhausted all the sugar present in the medium in 10 hr (Fig. 3c), with the highest value of maximum consumption rate of 1.40 g⋅L^−1^⋅hr^−1^, followed by *Sc. stipitis*, which was able to consume all the xylose in 18 hr at a rate of 1.17 g⋅L^−1^⋅hr^−1^ (Fig. 3a). In comparison to the aforementioned, the consumption rates of *Yamadazyma* sp., *N. liquefaciens, M. guilliermondii, P. tannophilus*, and *Meyerozyma* sp. under oxygen sparging were intermediate (Table 3), ranging from 0.75 to 0.45 g⋅L^−1^⋅hr^−1^ (listed in decreasing order). *Kluyveromyces marxianus* used xylose at the lowest rates in the aerated reactor, with a value of 0.39 g⋅L^−1^⋅hr^−1^. In terms of substrate consumption under oxygen provision, all strains were able to utilize between 90% and 100% of the pentose sugar, with the exception of *P. tannophilus*, which consumed about 70% of the substrate in 24 hr. Although the xylose consumption rate decreased for most of the strains in the non-oxygen-sparged reactors (70% and 40% HS conditions), *Sp. passalidarum* continued to be the yeast that consumed the total amount of xylose the fastest, both in the 70% and in the 40% HS setups (r_s_ of 0.70 and 1.00 g⋅L^−1^⋅hr^−1^, respectively). Interestingly, fermentation conditions had no significant effect on maximum xylose consumption rates of *Meyerozyma* sp., which utilized the sugar at approximately similar rates in aerated 70% and 40% HS reactors, although half of the xylose was used in the latter condition (Table 3).

In contrast, the maximum rates of xylose utilization of other strains, including *Sc. stipitis, N. liquefaciens, Yamadazyma* sp., and *M. guilliermondii*, decreased gradually as the stringency of oxygen supply increased from aerated reactors to 70% HS to 40% HS (Table 3 and Supplementary Fig. S1). These changes in xylose consumption were accompanied by a decrease in the maximum specific growth rate of the strains (Table 2). However, there was a marked contrast in the rate of sugar consumption with *Sc. stipitis* when oxygen was provided, but no significant difference was observed among the non-aerated reactors (Fig. 3a). The results also showed that the decrease in the available headspace (40% HS condition) most affected the utilization rates and biomass growth of *M. guilliermondii, N. liquefaciens*, and *Yamazyma* sp., which had the poorest r_s_ values among all the strains investigated (0.10, 0.09, and 0.06 g⋅L^−1^⋅hr^−1^, respectively). Furthermore, incomplete xylose consumption within 24 hr was noted for these strains. Particularly, for *P. tannophilus*, aeration increased the rates of xylose utilization by almost three times when compared to the two non-aerated cultures (70% and 40% HS). However, the difference in the HS of the reactors had no significant effect on the r_s_ values, but it did alter the amount of xylose used, with lower consumption in the 40% HS condition (Table 3). Aeration did not improve the maximum consumption rates of *K. marxianus* compared to the 70% HS condition, and the impact of oxygen on xylose utilization by this strain was less marked than the others, as *K. marxianus* had the lowest value of r_s_ under higher levels of oxygenation (Table 3).

When comparing the performance of the naturally xylose-assimilating yeasts with *S. cerevisiae* TMB3400, the engineered strain revealed the particularity of consuming xylose at similar rates under aerated and 40% HS conditions (Fig. 3e). Nevertheless, in the 70% HS reactor the strain utilized the sugar at a slower rate (Table 3). The *S. cerevisiae* TMB3400 strain carries the two genes from *Sc. stipitis* that encode for the XR and XDH enzymes required for xylose catabolism (Wahlbom et al., 2003). In both aerated and 70% HS cultures, *Sc. stipitis* used xylose faster and showed higher r_s_ values compared to the recombinant strain, but the opposite pattern was observed under more oxygen-restricted conditions (40% HS) (Table 3).

Overall, *Sp. passalidarum* and *Sc. stipitis* demonstrated robust xylose consumption, utilizing almost all the available sugar (ΔS ranging from 85% to 100%) at the end of the fermentation period (24 hr) across all three conditions employed (see Supplementary Fig. S1). Conversely, for the other native yeasts, the capacity to consume xylose was considerably reduced as a consequence of the narrowing of the reactor headspace (i.e. the decreased levels of oxygen). Specifically, in the 40% HS condition, all strains except *Sp. passalidarum, Sc. stipitis*, and *S. cerevisiae* TMB3400 showed the lowest percentage of xylose usage, with values below 55% of the initial sugar (Table 3).

These findings highlight the positive impact of aeration in stimulating xylose consumption to different extents and underscore the importance of the presence of oxygen in the media for efficient xylose catabolism. However, this effect is more pronounced in certain yeast species.

### Ethanol and Xylitol Production

The production of ethanol and/or xylitol, the main metabolites of xylose fermentation, was strongly affected by both the yeast species considered and the three reactor configurations employed. It has been demonstrated that the metabolism of xylose is conditioned by the oxygen availability in the media, with the carbon flux toward biomass or product formation being determined by the oxygen levels. Our findings indicate that modifying the working volume of the reactor impacted the fermentation profile, affecting the yields of ethanol and xylitol, most likely due to changes in the oxygenation conditions. High cell densities were obtained under high oxygen levels (aerated reactor) as shown previously (Table 2). This suggests that under such conditions, the selected yeasts metabolized the xylose through the oxidative pathway and respiration, and that excessive oxygen supply limited ethanol production. However, there was an exception observed with *Sp. passalidarum*, which produced ethanol during aerated culture (Table 4). This implies a possible reduced sensitivity of *Sp. passalidarum* to oxygen levels or a lowered dependence on oxygen for fermentative xylose metabolism leading to ethanol production. Regardless of which explanation accounts for this behavior, the importance of this finding cannot be overstated, as control of aeration levels in industrial processes is frequently a bottleneck when establishing robust bioprocesses.

**Table 4.** Ethanol and Xylitol Yields From Xylose Fermentation Measured in Three Different Culture Conditions

	**Ethanol yield [Y_e/x_ (g_ethanol_ g_xylose_ ^−^^1^)]**			**Xylitol yield [Y_xyl/x_ (g_xylitol_ g_xylose_ ^−^^1^)]**		
**Yeast strain**	**Aerated**	**70% headspace**	**40% headspace**	**Aerated**	**70% headspace**	**40% headspace**
*Scheffersomyces stipitis*	0.01	0.22	0.49	n.d.	0.07	0.02
*Pachysolen tannophilus*	n.d.	0.07 (24 hr)	0.42 (24 hr)	0.03	0.02	0.04
*Spathaspora passalidarum*	0.12	0.40	0.50	0.01	n.d.	n.d.
*Kluyveromyces marxianus*	0.01	0.14	0.16	0.01	0.11	0.16
*Naganishia liquefaciens*	n.d.	0.18	0.27	0.38	0.12	0.01
*Yamadazyma* sp.	n.d.	0.23	0.41	0.17	0.18	0.19
*Meyerozyma guilliermondii*	n.d.	0.04	0.04	0.23	0.15	0.10
*Meyerozyma* sp.	n.d.	0.01	0.01	0.47	0.46	0.46
*Saccharomyces cerevisiae* TMB3400	0.04	0.30	0.39	n.d.	0.04	0.14

*Note.* Ethanol yield values were calculated at the time of maximum ethanol production (in brackets), xylitol yields at the end of fermentation (24 hr). n.d. = not detected.

**Fig. 4.**
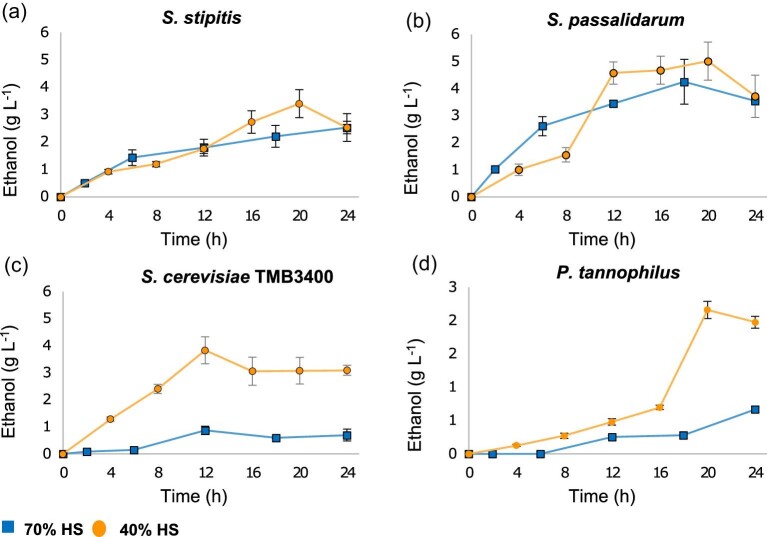
Ethanol production from xylose over time of the best xylose-fermenting yeast species in non-aerated reactors with 70% and 40% headspace. *Scheffersomyces stipitis* (a), *Spathaspora passalidarum* (b), *Saccharomyces cerevisiae* TMB3400 (c), and *Pachysolen tannophilus* (d) exhibited the highest ethanol titers and productivities among the nine xylose utilizers strains studied.

Fermentation assays in 70% and 40% HS yielded the highest amounts of ethanol by *Sc. stipitis, Sp. passalidarum, P. tannophilus*, and *S. cerevisiae* TMB3400, which exhibited the highest fermentation potential in terms of yields and productivity among the yeast strains examined (Table 4 and Fig. 4). Ethanol production was moderate in *Yamadazyma* sp., *N. liquefaciens*, and *K. marxianus*, giving intermediate yields with the poorest productivities, between 0.05 and 0.07 g_ethanol_ L^−1^⋅hr^−1^ (see Supplementary Fig. S2). However, the low levels of xylose consumption, which were less than 40%, together with the lowest ethanol concentrations, limit the interest in the aforementioned fermentative behavior.

For the xylose-fermenting strains, the 40% HS condition increased the formation of ethanol and the greatest yields were obtained (Table 4), which is consistent with the poor production of biomass and the most restricted oxygen environment among the three conditions tested. Within the native ethanol-producing strains, *Sp. passalidarum* and *Sc. stipitis* were the most efficient in xylose conversion to ethanol, reaching a maximum yield of 0.50 and 0.49 g_ethanol_ g_xylose_^−1^ in 40% HS with a productivity of 0.25 and 0.17 g_ethanol_ L^−1^⋅hr^−1^, respectively. Several studies confirm the distinguished fermentative capacity of *Sp. passalidarum* under oxygen-limited and anaerobic cultivations on d-xylose (Veras et al., 2017), while others highlight the good performance of *Sc. stipitis*, the most investigated non-conventional yeast species in terms of xylose fermentation (Ferreira et al., 2011). Our study also demonstrates that these species have the greatest potential for xylose fermentation under specific cultivation conditions, and ethanol yields close to the theoretical maximum (0.51 g_ethanol_ g_xylose_^−1^) were achieved under non-strictly anaerobiosis. Notably, the most effective configuration that maximized ethanol production by both strains was a flask-to-medium volume ratio (V_flask_:V_medium_) of 1.67 (40% HS) at a temperature of 35 °C. This is consistent with Su et al. (2015), where a similar V_flask_:V_medium_ ratio was used to achieve the lowest aeration condition that resulted in the highest ethanol yield (0.45 *g*⋅g^−1^) by *Sp. passalidarum*.

Although the reactor configuration altered the ethanol produced for all native fermenter yeasts, the most notable effect was observed for *P. tannophilus* (Fig. 4d), as the ethanol yield for this strain increased from 0.07 in 70% HS to 0.42 g_ethanol_ g_xylose_^−1^ in 40% HS, almost six times in a period of 24 hr. These data imply that the fermentative capacity of the strain is highly sensitive to the level of oxygenation and that minimal aeration is needed to ferment xylose with higher yields. When comparing the fermentation efficiency of *P. tannophilus* with that of *Sp. passalidarum* and *Sc. stipitis*, lower productivity (0.11 g_ethanol_ L^−1^⋅hr^−1^) and yield were detected.

**Fig. 5.**
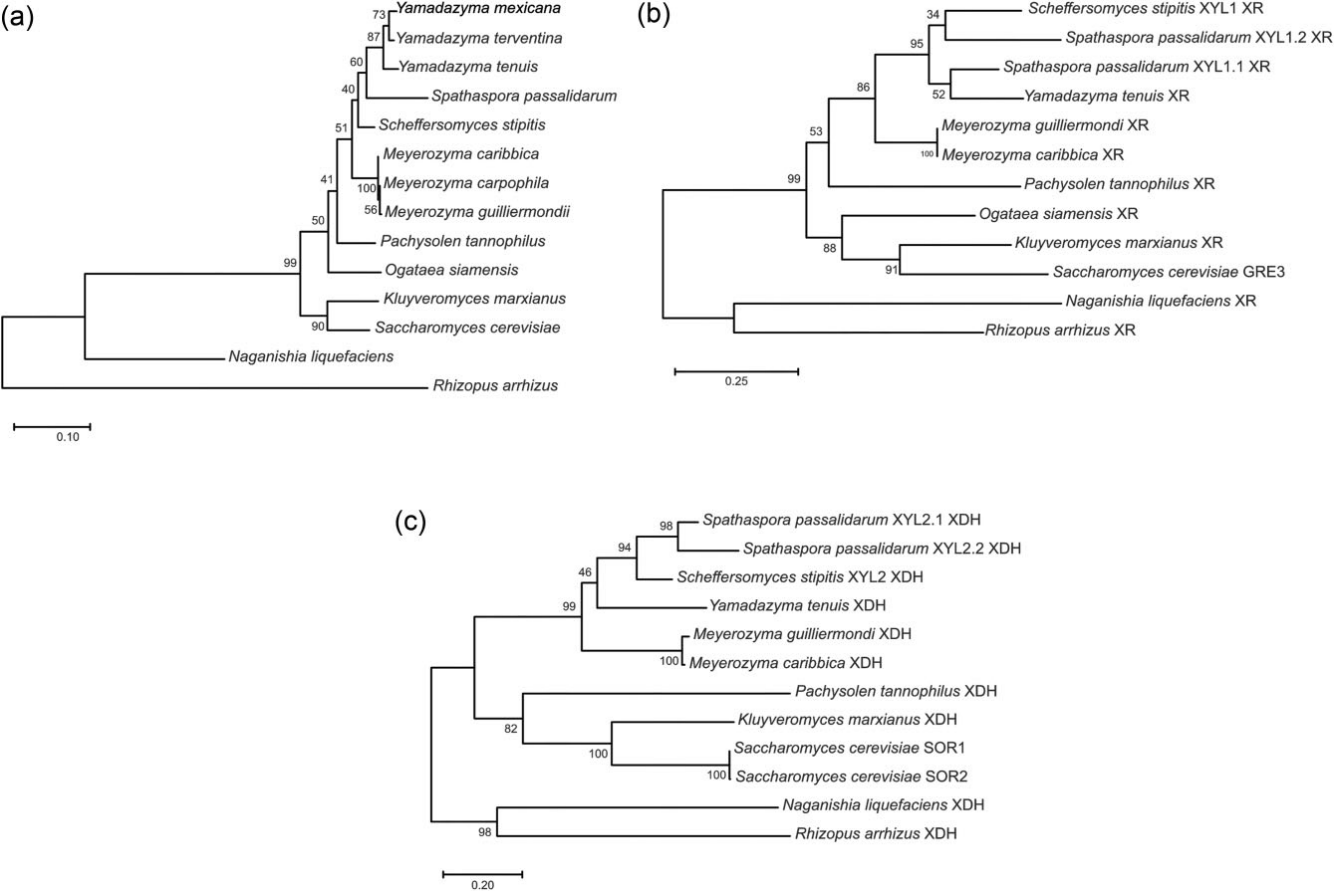
Phylogenetic tree of xylose-consuming yeasts. Evolutionary analysis was based on the DNA sequences of internal transcribed spacer (ITS)/D1-D2 (a) and the amino acid sequences of xylose reductase (XR) (b) and xylose dehydrogenase (XDH) (c) enzymes. Bootstrap values obtained from 1,000 repetitions are indicated on all branches. *Rhizopus arrhizus* was selected as the outgroup.

The engineered industrial *S. cerevisiae* TMB3400 strain was selected to compare the fermentative potential of naturally xylose-fermenting yeasts with a modified *S. cerevisiae* strain optimized for xylose fermentation (Wahlbom et al., 2003). Alcoholic fermentation with *S. cerevisiae* TMB3400 gave a lower ethanol yield (0.30 g_ethanol_g_xylose_^−1^) than with *Sp. passalidarum* in 70% HS (0.40 g_ethanol_g_xylose_^−1^) and a slightly higher one than that obtained with *Sc. stipitis* (0.22 g_ethanol_ g_xylose_^−1^). Even though the ethanol yield was higher in *S. cerevisiae* TMB3400 than in *Sc. stipitis* in the mentioned condition, the latter consumed more xylose and produced almost three times the amount of ethanol. When the engineered strain was cultivated at 40% HS, the fermentative performance in terms of yield and productivity (0.39 g_ethanol_g_xylose_^−1^ and 0.19 g_ethanol_ L^−1^ hr^−1^) was superior to the 70% HS condition, but inferior to *Sp. passalidarum* and close to *Sc. stipitis* (Fig. 5). Nevertheless, *Sc. stipitis* was found to yield more ethanol than the recombinant *S. cerevisiae* strain, which accumulated more xylitol than the native fermenter strains. However, the time of maximum ethanol production was shorter for this strain than for the native xylose-fermenting species (Table 4, 40% HS condition). The impact of the XR and XDH enzymes from *Sc. stipitis* on the genetic background of *S. cerevisiae* is noteworthy. *Scheffersomyces stipitis*, which is a Crabtree-negative yeast, is clearly sensitive to oxygen levels. An increase in oxygen availability from 40% to 70% HS significantly reduces the ethanol yield by almost half compared to the most unfavorable aeration condition. In contrast, *S. cerevisiae*, a Crabtree-positive yeast with a genetic background favoring fermentation, experiences a more modest decrease in ethanol yield of about 20% when transitioning from 40% to 70% HS. This underscores the resilience of *S. cerevisiae* to oxygen levels, maintaining its fermentative metabolism even under conditions that particularly affect the xylose pathway in *Sc. stipitis*.

The *K. marxianus* strain isolated in this work had a mild xylose fermentation ability and gave intermediate ethanol yields under both 70% and 40% HS (0.14 and 0.16 g_ethanol_ g_xylose_^−1^) with a minimum productivity of 0.06 g_ethanol_ L^−1^⋅hr^−1^. The differences in the fermentation conditions did not substantially influence the ethanol production by this strain compared to the other yeasts under investigation. Regarding what is known about xylose metabolism in *K. marxianus*, different strains of this species are capable of directing xylose to ethanol with varying yields. However, the xylose fermentative efficiency and ethanol productivity remain lower in comparison to other ethanol-producer species, such as *Sp. passalidarum* and *Sc. stipitis*. Other *K. marxianus* strains only assimilate xylose but lack ethanolic fermentation capacity (Nitiyon et al., 2016).

*Spathaspora passalidarum, Sc. stipitis, P. tannophilus*, and *S. cerevisiae* TMB3400 produced negligible or only minimal amounts of xylitol under aeration. On the contrary, *N. liquefaciens, Yamadazyma* sp., *M. guilliermondii*, and *Meyerozyma* sp. were the species that mainly produced xylitol during xylose utilization in aerated cultures, and no ethanol was detected in this condition (Table 4). *Naganishia liquefaciens* and *Meyerozyma* sp., both isolates from tannery wastewater, showed the highest xylitol yields (0.38 and 0.47 g_ethanol_ g_xylose_^−1^, respectively) and, at the same time, the lowest values of biomass formation when oxygen was supplied (Table 2). It is generally assumed that xylitol accumulates as a result of disparity in cofactor utilization between the XR and XDH enzymes when yeasts catabolize xylose via the oxidoreductive pathway. This cofactor imbalance occurs mainly in fully anaerobic cultures and under oxygen limitation, where the regeneration of nicotinamide adenine dinucleotide (NAD^+^) used by XDH is limited. In our results, xylitol was produced in significant quantities in aerated cultures where oxygen supply should be sufficient to oxidize nicotinamide adenine dinucleotide phosphate hydrogen (NADPH) and provide NAD^+^ for xylitol to xylulose conversion. This suggests that there may be other reasons for xylitol accumulation in certain yeast species in addition to the redox imbalance caused by different cofactor needs. Further experiments will be necessary to elucidate this matter. Among the xylitol-producing yeasts, *Meyerozyma* sp. accumulated the largest amount of xylitol under the three conditions studied, and this strain did not appreciably reduce xylitol formation as an effect of limiting oxygen in the reactor setups. A similar behavior can be observed with the *Yamadazyma* strain. In contrast, *N. liquefaciens* showed a significant reduction in xylitol production with decreasing aeration (70% HS) and almost no xylitol was produced in the most restricted condition (40% HS) (Table 4). Similarly, but less markedly, xylitol production by *M. guilliermondii* decreased almost twofold with the increase in oxygen limitation. Oppositely, a more oxygen-restricted environment favored xylitol generation by *K. marxianus* and *S. cerevisiae* TMB3400.

In general, our findings reveal significant variations in xylose consumption, metabolic by-products, and fermentation efficiency across different species, with the majority of strains displaying a clear dependence on oxygen levels. However, certain strains, such as *Sp. passalidarum* and *Meyerozyma* sp., were less sensitive to fluctuations in oxygen levels. This trait positions them as suitable candidates for the development of consolidated bioprocesses, whether for ethanol or xylitol production, using xylose-rich feedstocks.

### Phylogenetic Relationship of d-Xylose-Consuming Yeasts

The majority of xylose-assimilating yeasts use a two-step enzymatic oxidoreductive process involving XR (EC 1.1.1.21) and XDH (EC 1.1.1.9) to metabolize d-xylose. Xylose reductase facilitates the reduction of d-xylose to xylitol, while XDH catalyzes the oxidation of xylitol to d-xylulose. Subsequently, d-xylulose is converted into d-xylulose 5-phosphate by xylulose kinase before entering the pentose phosphate pathway. The sequences of XR and XDH enzymes are highly conserved among different microorganisms, suggesting their evolutionary and functional importance. In an attempt to identify the phylogenetic relationships between these enzymes and the evolution of xylose metabolic pathway in the different yeast genera selected for this study, phylogenetic trees were constructed using the ITS/D1-D2 sequences (Fig. 5a) and the amino acid sequences of the XR and XDH enzymes (Fig. 5b and 5c, respectively).

The phylogenetic analyses revealed that *Yamadazyma* species were more closely related to *Sp. passalidarum* and together with *Sc. stipitis* formed a clade that was separated from the *Meyerozyma* clade (Fig. 5a). Within the *Meyerozyma* group, all analyzed species were clustered together. Notably, *N. liquefaciens*, a basidiomycete yeast, differed from all other ascomycete yeasts and was grouped separately in a subtree (Fig. 5a). Trees constructed with XR and XDH amino acid sequences (Fig. 5b and c) showed similar phylogenetic relationships to that constructed with ITS sequences. In natural xylose-metabolizing yeasts, the *XYL1* gene encodes the XR enzyme and the *XYL2* gene encodes the XDH enzyme. *Spathaspora passalidarum* strains have two *XYL1* genes, responsible for encoding two XRs with different cofactor preferences, the first for nicotinamide adenine dinucleotide hydrogen (NADH) (*XYL1.1*) and the second (*XYL1.2*) with increased NADH affinity (Cadete et al., 2016). Two homologs of the *XYL2* gene (*XYL2.1* and *XYL2.2*) have also been found in this species. The enzymes resulting from *Sp. passalidarum XYL1.1* and *XYL2.1* genes resemble those found in other yeasts.

Our data show that XR and XDH from *Meyerozyma* species, known for their propensity to produce xylitol, were grouped together and separated from XR from *Yamadazyma tenuis* (formerly *Candida tenuis*), *Sp. passalidarum*, and *Sc. stipitis*. When comparing the enzymatic amino acid sequences of both *Meyerozyma* strains with other yeast species, the enzymes from *M. guilliermondii* and *M. caribbica* were the most identical (see Supplementary Table S2), indicating a similar divergence within the XRs and XDHs of this clade. The *Sp. passalidarum, Sc. stipitis*, and *Y. tenuis* species have previously been placed in one group and classified as xylose fermenters, but the ability of *Y. tenuis* to convert xylose into ethanol has been questioned (Veras et al., 2017). Considering the fermentation results presented earlier, the observed variability in the literature regarding the fermentative potential of *Yamadazyma* strains may be attributed not only to species divergence, but also to differences in oxygen levels between assays performed by different research groups.

Although *P. tannophilus* is also capable of xylose fermentation, its XR and XDH enzymes were positioned separately from the aforementioned xylose-fermenting species (Fig. 5b and c). It is worth noting that in our phylogenetic outcomes we are looking at global enzyme sequence alignments rather than specific domains or active sites that may share particular features within xylose-to-ethanol producing species. The ability to consume and ferment xylose should not only be correlated with the phylogenetic placement of a given species in a particular clade or group and/or with the presence of XR and XDH genes. In fact, it has been shown that some species encoding this set of enzymes were unable to assimilate xylose. Therefore, additional genetic factors and other gene clusters independent from XR and XDH should also be considered.

## Discussion

Many native xylose-assimilating yeasts have been mainly associated with decaying wood, beetle guts, tree bark, fruits, and soil samples (Cadete et al., 2016; Nguyen et al., 2006). In our study, a comparative analysis of xylose utilization by different yeast species was performed, including most known xylose-fermenting strains, *Sp. passalidarum* and *Sc. stipitis*, as well as novel isolates from less common resources, such as tannery wastewaters. The strains isolated from these environmental samples belonged to the genera *Naganishia* and *Meyerozyma.* Other strains of the genera *Yamadazyma, Meyerozyma*, and *Kluyveromyces*, found in tree bark, wine spoilage, and dairy wastewater, respectively, were also characterized.

Because xylose metabolism in non-conventional yeasts is closely linked to oxygen availability, the fermentation performance of the strains was evaluated in three distinct reactor setups. The importance of establishing differential aeration conditions to better understand the influence of oxygen on the xylose catabolic capacity of natural xylose-assimilating yeast has also been highlighted by other authors (Barros et al., 2024). In our study, the variations in oxygenation were reflected in the amount of biomass generated, the maximum rates of xylose consumption and biomass formation, and the tendency of the yeast species to produce xylitol and/or ethanol. In general, higher aeration promoted cell growth, whereas this condition had the opposite effect on ethanol formation, and the conversion of xylose to ethanol was strongly favored in the most oxygen-restricted condition (40% HS). Similar effects of oxygen on xylose growth and fermentation have been demonstrated in several studies, although the aerobic and oxygen-limited cultures were achieved differently (Su et al., 2015; Veras et al., 2017). Reducing the oxygen levels progressively lowered the maximum rates of biomass formation and substrate consumption, although the impact of oxygenation on these parameters was variable among the yeasts. While the specific growth rates decreased for all strains from aerated to non-aerated cultures, the difference in xylose utilization cannot be translated into a single pattern, as it was mainly dependent on both the yeast species and the condition studied. For instance, some species, particularly the ones that produced appreciable amounts of xylitol when air was provided, such as *Yamadazyma* sp., *M. guilliermondii*, and *N. liquefaciens*, had the lowest values of xylose consumption rates and amount of sugar used in the most oxygen-restricted cultures compared to the most aerated one. For these species, excluding *Meyerozyma* sp., aeration remarkably enhanced xylose consumption. Similarly, Barros et al. (2024) reported that strains, which mainly accumulate xylitol as a by-product, *M. guilliermondii, M. caribbica*, and various *Candida* species, displayed increased xylose consumption with higher aeration. These closely related species were found to be highly dependent on oxygen for xylose utilization. Instead, our *Meyerozyma* sp. strain had moderate maximum xylose consumption rates, which were maintained throughout the three conditions used. In contrast, of the native xylose-utilizing yeasts, *Sp. passalidarum* and *Sc. stipitis*, the two with the highest fermentative performance were the most efficient at consuming xylose at the lowest aeration. Under this condition, *Sp. passalidarum* displayed the fastest utilization of 100% of the sugar. Cadete et al (2016) found higher xylose consumption rates and usage in *Sp. passalidarum* strains than in xylitol-producing *Spathaspora* species when cultivated under more severe oxygenation conditions. The outstanding ability of *Sp. passalidarum* to use and ferment xylose under restricted oxygen provision and anaerobiosis has been attributed to the preferential use of NADH by the XR enzyme.

The differing patterns of xylose usage and metabolite accumulation by native yeasts can be partially explained by variations in the initial reactions of xylose catabolism. In certain yeast species, xylose is first reduced to xylitol by an XR that prefers or solely uses NADPH, while in other species, an XR that prefers NADH for the enzymatic reaction is present. In the second metabolic step, the resulting xylitol is either secreted or oxidized by an NAD^+^-dependent XDH. When the XR inclination is toward NADPH, a cofactor imbalance is created between the two oxido-reductive reactions, leading to the accumulation of xylitol. Insufficient oxygen supply causes overproduction of NADH, impairing the regeneration of NAD^+^ for the XDH reaction. This prevents the flux toward the conversion of xylitol into xylulose. In contrast, xylose metabolism via an XR capable of accepting NADH as a cofactor, as seen in *Sp. passalidarum*, overcomes the redox imbalance. This allows the fermentation of xylose to ethanol under limited oxygen conditions. The results of our fermentation study revealed that switching from aeration to a higher oxygen restriction resulted in a greater ethanol production by *Sp. passalidarum, Sc. stipitis, P. tannophilus*, and *S. cerevisiae* TMB3400, with minimal to no xylitol yields, with the exception of the engineered *S. cerevisiae* strain. An important observation is that the *GRE3* gene, which encodes an NADPH-dependent aldose reductase, has been shown to have XR activity, which contributes to xylitol formation in *S. cerevisiae* strains. Consequently, inactivation of *GRE3* in laboratory and industrial strains has been implemented as a strategy to reduce xylitol formation (Romaní et al., 2015; Träff et al., 2001). The isolates of *K. marxianus, Yamadazyma* sp., and *N. liquefaciens* also generated ethanol, albeit to a much lesser extent and with limited productivities. For the less explored xylose utilizers strains, *N. liquefaciens* and *Yamadazyma* sp., the detected xylitol yields exceeded those obtained by the best ethanol-producer species. The closely related strains of *Meyerozyma* sp. and *M. guilliermondii* could produce xylitol with substantial yields and hardly any ethanol was measured under any condition. In the literature, species belonging to the *Candida* and *Meyerozyma* genera are recognized as efficient xylitol producers and the NADPH dependence of XR appears to be a common feature among these yeasts (Gurpilhares et al., 2008; Kim et al., 2019).

Although there seems to be a correlation between cofactor preference and the generation of ethanol and/or xylitol, as XR-preferring NADH correlates with fermentative ability (Cadete et al., 2016) and NADPH dependence prevails in xylitol production, this cannot be the sole reason why some species perform better or produce more of a metabolite than others. It should be also taken into account the differential expression of genes involved in xylose metabolism in each xylose-assimilating yeast. Our findings revealed that *Meyerozyma* sp. and *N. liquefaciens* strains exhibited more xylitol production when aeration was supplied. In this condition, XDH should get more cofactors to oxide the xylitol as the levels of NAD^+^ increase. Therefore, these findings suggest that factors other than the cofactor imbalance may contribute to the production of xylitol in certain yeasts. Barros et al. (2024) observed the accumulation of xylitol under high oxygenation in some species of the *Scheffersomyces* genus, indicating that this pattern may be limited to certain xylose-assimilating strains and may not be generalizable to others.

*Naganishia liquefaciens* is a not well-known xylose-consuming yeast. The ITS sequence alignment showed that this species is not closely related to any of the genera studied here. In fact, it was grouped separately from all the species analyzed. The same output was observed when analyzing the relationships of the xylose metabolic enzymes, XR and XDH, which displayed the lowest percentage of identity (values below 50%) with enzymes of the other genera. Our data indicate that the *Naganishia* strain isolated from tannery wastewater is a more efficient producer of xylitol than of ethanol. Other studies have highlighted that *Naganishia* species have a high metabolic capacity for producing industrially relevant enzymes, such as proteases and xylanases (Scorzetti et al., 2000). Lara et al. (2014) have characterized the growth performance of *N. diffluens* (formerly *Cryptococcus diffluens*) on xylose, but there is currently no data available on the consumption of xylose or the by-products formed by *Naganishia* strains. Combining our results with further screening of the ability to hydrolyze lignocellulosic substrates by secreting enzymes, *Naganishia* species could be considered as potential candidates in the development of CBP processes.

The yeasts studied here are classified as Crabtree-negative and differ from the Crabtree-positive *S. cerevisiae*. In native species that assimilate xylose, the transition between respiratory and fermentative metabolism is markedly dependent on oxygen, with the oxidative pathway being favored under higher oxygen levels and the fermentative capacity being reduced. Ethanol production in these yeasts is then triggered by restricting the oxygen input (Bonan et al., 2021). This trend correlates with the behavior of the xylose fermenter strains evaluated in this study, which predominantly channeled the sugar into the fermentative pathway in non-aerated reactors. This pattern was also noticeable in the recombinant strain *S. cerevisiae* TMB3400. When aerated and xylose was used as the carbon source, this engineered strain showed no fermentation ability, opposite to what occurs in *S. cerevisiae* strains cultivated under high glucose and aerobiosis. This is consistent with published results by Wahlbom et al. (2003). If the performance of *S. cerevisiae* TMB3400 is compared to that of wild xylose-fermenting strains (*Sp. passalidarum, Sc. stipitis*, and *P. tannophilus*) in terms of ethanol yields, *S. cerevisiae* TMB3400 had the lowest value in the most restricted oxygen reactor, but a much higher xylose consumption than that of *P. tannophilus* and a comparable one to that of *Sc. stipitis*. The improved performance of the recombinant strain should not solely be attributed to the insertion of *Sc. stipitis* genes. This is because the strain underwent random mutagenesis and adaptive laboratory evolution, leading to changes in the expression of specific genes.

In our comparative study, *Sp. passalidarum* confirmed its status as the best xylose-fermenting strain. As it exhibited the highest ethanol yield and productivity and the highest xylose consumption rate among the three conditions tested, future research should focus on understanding the metabolic and sensing networks that regulate xylose metabolism and fermentation in this yeast.

Last but not least, it is worth mentioning the apparent resilience of *Sp. passalidarum* to oxygen levels in terms of ethanol production, as it was the yeast that maintained the highest yields throughout the different experimental setups. Furthermore, an intriguing observation was the low biomass yield under conditions of limited aeration, which coincided with the highest ethanol yields. So, this yeast can be visualized as a suitable partner for *Saccharomyces* strains in the development of CBP based on mixed inocula. Future work could focus on the metabolism of *Sp. passalidarum* in mixtures with different sugars, ethanol tolerance, and metabolic compatibility in a scenario of mixed fermentation with industrial *Saccharomyces* for the production of 2G-bioethanol from lignocellulosic biomass.

## Conclusions

The analysis of sugar consumption profiles, kinetic parameters, and yeast metabolic capacities proved to be attractive when considering the development of a bioprocess toward the production of value-added compounds. We conducted a comparative study involving natively xylose-assimilating yeasts for xylose usage and metabolic potential under identical experimental conditions. The native species assessed belonged to the genera *Yamadazyma, Meyerozyma, Naganishia, Kluyveromyces, Pachysolen, Scheffersomyces*, and *Spathaspora*, and their performance was also contrasted with that of an engineered strain of *S. cerevisiae*. Our research emphasizes the importance of characterizing xylose consumption under different oxygen conditions, as xylose metabolism was clearly affected to varying degrees by oxygenation in diverse yeast species. Overall, we found a positive correlation between higher xylose consumption and higher oxygen supply, but this effect was more evident for some strains, which tended to produce xylitol rather than ethanol, such as *Yamadazyma* sp., *M. guilliermondii*, and *N. liquefaciens*. For others, including *Sp. passalidarum* and *Sc. stipitis*, aeration stimulated xylose usage to a considerable minor degree and both strains were the best xylose consumers and fermenters under more oxygen-restricted conditions.

## Supplementary Material

kuae023_Supplemental_File
