# Supplementary material for: Exploring xylose metabolism in non-conventional yeasts: kinetic characterization and product accumulation under different aeration conditions
Source: J Ind Microbiol Biotechnol. 2024 Jun 27;51:kuae023. doi: 10.1093/jimb/kuae023 (PMC11247345; doi:10.1093/jimb/kuae023)
Supplement: kuae023_Supplemental_File [file kuae023_supplemental_file.docx]

**Supplementary Material**

**Exploring xylose metabolism in non-conventional yeasts: kinetic characterization and product accumulation under different aeration conditions**

Bruna C. Bolzico^1,2^, Sofia Racca^1^, Jorge N. Khawam^1^, Rodrigo J. Leonardi^1,2^, Ariel H. Tomassi^1^, Lisandro G. Seluy^1,2^, Maria T. Benzzo^1^ and Raul N. Comelli^1,2^*

**^1^**Grupo de Procesos Biológicos en Ingeniería Ambiental (GPBIA), Facultad de Ingeniería y Ciencias Hídricas (FICH), Universidad Nacional del Litoral (UNL), Argentina.

^2^Consejo Nacional de Investigaciones Científicas y Técnicas (CONICET), Ciudad Universitaria CC 242 Paraje El Pozo, Santa Fe 3000, Argentina.

* Corresponding author

E-mail: rcomelli@fich.unl.edu.ar

**Supplementary Material Tables**

**Table S1.** Accession numbers of amino acid sequences used in the phylogenetic study.

| **Species** | **XR** | **XDH** |
| --- | --- | --- |
| *Sc. stipitis* | [XP_001385181.1](https://www.ncbi.nlm.nih.gov/protein/XP_001385181.1?report=fasta) | [XP_001386982.1](https://www.ncbi.nlm.nih.gov/protein/XP_001386982.1?report=fasta) |
| *Sp. passalidarum* | [ALP00843.1](https://www.ncbi.nlm.nih.gov/protein/ALP00843.1?report=fasta) (*XYL1.1*)  [XP_007375534.1](https://www.ncbi.nlm.nih.gov/protein/XP_007375534.1?report=fasta)  (*XYL1.2*) | [XP_007373266.1](https://www.ncbi.nlm.nih.gov/protein/XP_007373266.1?report=fasta) (*XYL2.1*)  [XP_007374048.1](https://www.ncbi.nlm.nih.gov/protein/XP_007374048.1?report=fasta)  (*XYL2.2*) |
| *Y. tenuis* | [O74237.1](https://www.ncbi.nlm.nih.gov/protein/O74237.1?report=fasta) | [XP_006689111.1](https://www.ncbi.nlm.nih.gov/protein/XP_006689111.1?report=fasta) |
| *Y. mexicana* | - | - |
| *Y. terventina* | - | - |
| *M. guilliermondii* | [ABB87187.1](https://www.ncbi.nlm.nih.gov/protein/ABB87187.1?report=genbank&log$=protalign&blast_rank=2&RID=ZHBKV2SY01R) | XP_001481963.2 |
| *M. carphophila* | - | - |
| *M. caribbica* | [AWX90327.1](https://www.ncbi.nlm.nih.gov/protein/AWX90327.1?report=fasta) | [QAV56481.1](https://www.ncbi.nlm.nih.gov/protein/QAV56481.1?report=fasta) |
| *P. tannophilus* | [P78736.1](https://www.ncbi.nlm.nih.gov/protein/P78736.1?report=fasta) | [ODV98336.1](https://www.ncbi.nlm.nih.gov/protein/ODV98336.1?report=fasta) |
| *O. siamensis* | [ACN78427.2](https://www.ncbi.nlm.nih.gov/protein/ACN78427.2?report=fasta) | - |
| *K. marxianus* | [ADV91498.1](https://www.ncbi.nlm.nih.gov/protein/ADV91498.1?report=fasta) | [ADW84693.1](https://www.ncbi.nlm.nih.gov/protein/ADW84693.1?report=fasta) |
| *S. cerevisiae* | [NP_011972.1](https://www.ncbi.nlm.nih.gov/protein/NP_011972.1?report=fasta)  (*GRE3*) | [NP_012693.1](https://www.ncbi.nlm.nih.gov/protein/NP_012693.1?report=fasta)  (*SOR1*) |
| *N. liquefaciens* | [GHJ89122.1](https://www.ncbi.nlm.nih.gov/protein/GHJ89122.1?report=genbank&log$=prottop&blast_rank=1&RID=ZSP089D5016) | [GHJ86911.1](https://www.ncbi.nlm.nih.gov/protein/GHJ86911.1?report=fasta) |
| *R. arrhizus* | [AHG97682.1](https://www.ncbi.nlm.nih.gov/protein/AHG97682.1?report=fasta) | [KAG0737167.1](https://www.ncbi.nlm.nih.gov/protein/KAG0737167.1?report=fasta) |

**Table S2. Percentage of identity of XR and XDH between xylose-assimilating species.**

| **Species** | **XR**  **(*XYL1*)** | **XDH**  **(*XYL2*)** |
| --- | --- | --- |
| *S. stipitis*  *S.passalidarum (XYL1.1*; *XYL2.1*) | 75.47% | 82.97% |
| *S. stipitis (XYL1*; *XYL2)*  *S.passalidarum (XYL1.2*; *XYL2.2*) | 74.13% | 77.75% |
| *S. stipitis*  *Y. tenuis* | 77.04% | 73.08% |
| *S. passalidarum* (*XYL1.1*; *XYL2.1*)  *Y. tenuis* | 80.82% | 71.98% |
| *S. passalidarum* (*XYL1.2*; *XYL2.2*)  *Y. tenuis* | 73.50% | 65.75% |
| *M. guilliermondii*  *M. caribicca* | 98.74% | 97.24% |
| *M. guilliermondii*  *Y. tenuis* | 70.98% | 68.04% |
| *P. tannophilus* |  |  |
| *S. stipitis* | 61.64% | 47.98% |
| *P. tannophilus*  *Y. tenuis* | 62.19% | 49.19% |
| *K. marxianus*  *S. cerevisiae* (*GRE3;SOR1*) | 66.97% | 66.29% |
| *N. liquefaciens*  *R. arrhizus* | 46.27% | 48.13% |

Percentage identity values at the protein level of the enzymes XR (encoded by *XYL1* genes) and XDH (encoded by *XYL2* genes). Yeast species are grouped for comparison as efficient ethanol producers (*S. passalidarum*, *S. stipitis*, and *P. tannophilus*), efficient xylitol producers (*M. guilliermondii*, *M. caribicca*), and less explored species (*Y. tenuis*, *N. liquefaciens*). XR: Xylose reductase; XDH: Xylitol dehydrogenase; *GRE3*: Aldose reductase; *SOR1*: Sorbitol dehydrogenase.

**Supplementary Material Figures**


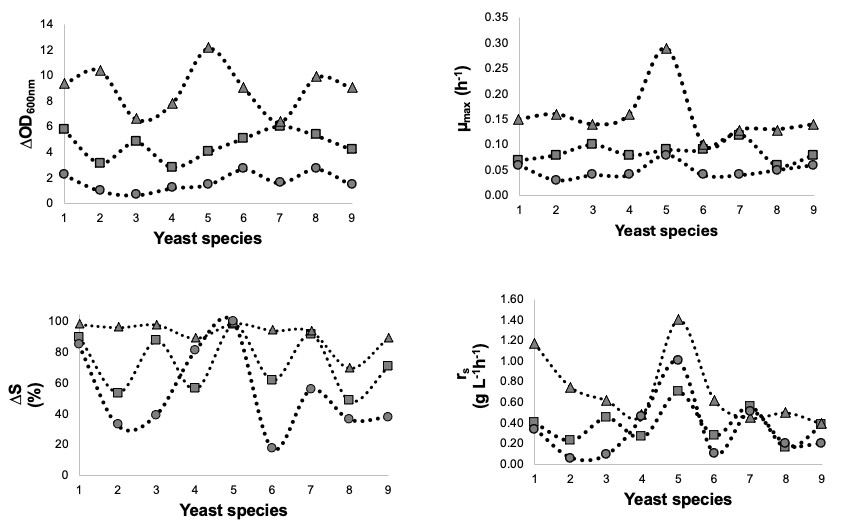


**Fig. S1. Comparison of the fermentation parameters for each individual yeast species in each of the three cultivation variants: aerated reactors (open triangle), 70% headspace (open square) and 40% headspace (open circle).**

*References:* the numbers 1 to 9 on the x-axis correspond to **1**, *Sc.stipitis*; **2**, *Yamadazyma* sp.; **3**, *N.* *liquefaciens*; **4**, *S. cerevisiae* TMB3400; **5**, *Sp.* passalidarum; **6**, *M. guilliermondii*; **7**, *Meyerozyma* sp.; **8**, *P. tannophilus* and **9**, *K. marxianus*. **r_s_**: volumetric xylose consumption rate; **µ_max_** : maximum specific growth rate, **ΔS**: net xylose consumption; **ΔOD_600nm_**: net optical density at 600 nm.


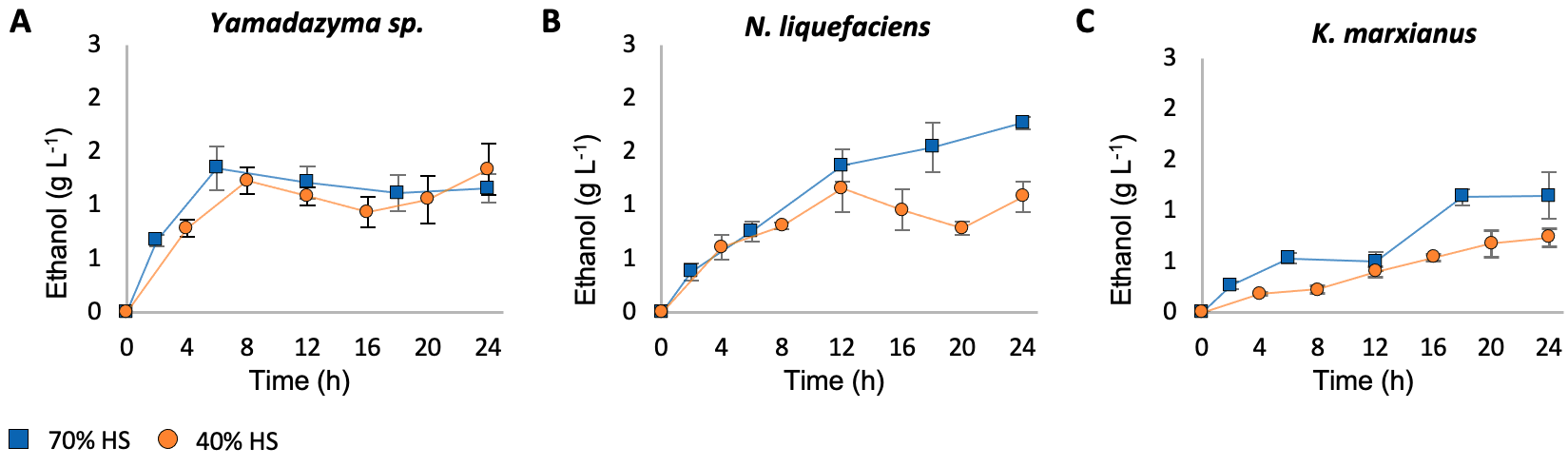


**Fig. S2. Ethanol accumulation over time for poor performing xylose fermentation yeast species.** (A) *Yamadazyma* sp., (B) *N. liquefaciens* and *K. marxianus* (C) in 70% (blue square) and 40% (orange circle) headspace reactors.
